# Topographic Outcomes in Keratoconus Surgery: Epi-on versus Epi-off Iontophoresis Corneal Collagen Cross-Linking

**DOI:** 10.3390/jcm11071785

**Published:** 2022-03-24

**Authors:** Pasquale Napolitano, Fausto Tranfa, Luca D’Andrea, Ciro Caruso, Michele Rinaldi, Alberto Mazzucco, Nicola Ciampa, Antonietta Melenzane, Ciro Costagliola

**Affiliations:** 1 Department of Medicine and Health Sciences “Vincenzo Tiberio”, University of Molise, 86100 Campobasso, Italy; napolitano.pasquale1989@gmail.com; 2 Department of Neurosciences, Reproductive Sciences and Dentistry, Eye Clinic, University of Naples “Federico II”, 80138 Naples, Italy; fausto.tranfa@unina.it (F.T.); mazzucco.alberto@libero.it (A.M.); nicolaciampa3@gmail.com (N.C.); antonietta.melenzane@unina.it (A.M.); ciro.costagliola@unina.it (C.C.); 3 Public Health Department, Università degli Studi di Napoli “Federico II”, 80138 Naples, Italy; 4 Corneal Transplant Center, Pellegrini Hospital, Via Portamedina alla Pignasecca, 41, 80127 Napoli, Italy; cirocarusoeye@gmail.com; 5 Department of Ophthalmology, University Della Campania Luigi Vanvitelli, 80138 Naples, Italy; michrinaldi@libero.it

**Keywords:** corneal collagen cross-linking, keratoconus, epi-on technique, epi-off technique, iontophoresis, cornea, pachymetry, thinnest point, CXL, OSDI

## Abstract

Background: Corneal collagen cross-linking (CXL) has become the gold standard for mild and moderate stages to stop the progression of keratoconus. We analyzed some corneal topography indices to compare iontophoresis epi-on and iontophoresis epi-off techniques throughout a two-year follow-up. Methods: A total of 64 eyes of 49 patients who underwent CXL were recruited. In 30 eyes the epi-off technique was performed, whereas the remaining 34 eyes were treated with the epi-on technique. All patients underwent a complete ophthalmologic examination that included CDVA, central and thinnest corneal thickness, Schirmer test I, TBUT test, and the Ocular Surface Disease Index. Results: In both groups, a significant improvement in visual function was recorded. No statistically significant differences between Kmax, Mean K, Flat K, Steep K values were found. Statistically significant differences (*p* < 0.05) between the epi-on and epi-off groups’ pachymetry before and after 24 months follow-up as well as between the epi-on and epi-off groups’ topographically thinnest point in the immediate post-surgery and 24 months after surgery were recorded. Conclusion: Our study highlighted that both techniques are valid in mid-term corneal stabilization. The advantage of the new iontophoresis epi-off cross-linking technique could be found in a faster imbibing time of the cornea, therefore reducing surgical times, with a lower risk of complications.

## 1. Introduction

Keratoconus (KC) is a corneal ectasia and diverse genetic, environmental, and biochemical factors have been associated with it. Increased systemic levels of pro-inflammatory factors, including interleukin-6, tumor necrosis factor-α, and matrix metalloproteinase-9 were found, suggesting that KC may have an inflammatory component [1]. Nevertheless, the etiopathological mechanisms are still not completely elucidated [2]. Keratoconus is characterized by the thinning of the corneal stroma, which leads to the cornea taking a conical shape, generating irregular astigmatism, and increasing the level of higher-order aberrations, with the consequent progressive deterioration of vision [2]. The typical onset of KC is puberty [3,4], with pediatric patients showing the highest rate and speed of progression [5]. In this light, prompt surgical intervention has been suggested in children and adolescents [6,7]. Corneal collagen cross-linking (CXL) has become the gold standard for mild and moderate stages to stop the progression of ectasia [6,8]. This technique aims to strengthen the stromal collagen by induction of collagen bonds activated by ultraviolet light. Thanks to the use of riboflavin-soaked cornea [9], the cross-links between corneal lamellae can increase the biomechanical strength and stability of the cornea or slow the progression of keratoconus. The standard Dresden cross-linking protocol has been shown to lead to keratoconus stabilization over a mid-term and long-term follow-up [10,11]. The standard corneal cross-linking procedure includes the removal of corneal epithelium to achieve adequate penetration of riboflavin into the stroma. Unfortunately, epithelial removal may be responsible for crosslinking-related complications, i.e., vision impairment, risk of infection, and postoperative pain [12]. In the attempt to avoid the side effects induced by epithelial debridement, a trans-epithelial cross-linking technique has been recently introduced [13,14,15].

For both techniques, the first step is represented by a corneal soak. This step is realized with or without epithelial debridement and requires about 30 min. Iontophoresis guarantees a faster delivery (5 min) of charged molecules into corneal stroma through a small electric current. The second step consists of the activation of penetrated riboflavin with ultraviolet A (UVA) light for about 9 min; the intensity of the UVA light source is 10 mW/cm^2^. The last step is the same for both techniques [10,15,16].

This study has aimed to analyze some corneal topography indices comparing, after corneal collagen crosslinking, iontophoresis epi-on (without epithelial debridement) and iontophoresis epi-off (with epithelial debridement) techniques throughout the two-year follow-up period.

## 2. Materials and Methods

A prospective interventional study, approved by the institution’s review board and in accordance with the Declaration of Helsinki, was conducted on patients affected by keratoconus, referred to the Unit of Ophthalmology of the University of Naples “Federico II” from January 2018 to December 2020. We included patients affected by KC and listed for CXL surgery according to the following criteria: (1) patients with progressive keratoconus (1 diopter increase in the steepest meridian during a 6-month observation period); (2) Stage 2 or 3 of keratoconus (Amsler–Krumeich classification); (3) Corrected distance visual acuity (CDVA) < 0.4 logMAR; and (4) central clear cornea and minimum corneal thickness of at least 450 μm.

We divided patients into two groups through the block randomization method of treatment depending on the treatment performed: CXL by iontophoresis with or without epithelium removal.

### 2.1. Surgical Procedure

Corneal cross-linking was performed by instilling dextran-free hypo-osmolar riboflavin containing benzalkonium chloride (Ricrolin+, SOOFT, Montegiorgio, Italy) on the cornea by iontophoresis to increase the permeability of the epithelium for 5 min.

A return electrode was placed on the skin of the frontal region; meanwhile, the corneal iontophoresis electrode was attached to the cornea through a vacuum adsorption device. The corneal electrode was filled with approximately 0.5 mL Ricrolin+ to fully immerse the stainless-steel mesh. To follow, the device was connected to a current generator (I-ON XL, SOOFT, Montegiorgio, Italy) with 1 mA current.

At the end of iontophoresis, a UVA light was then focused on the apex of the cornea, 10 mW/cm^2^ (UV-X 2000; IROC Innocross AG, Zug, Switzerland) for 9 min. During irradiation, drops of balanced solution were applied to the cornea every 1 min to keep moisture to avoid complications.

When the epi-off CXL technique was performed, the corneal epithelium was mechanically removed with a blunt spatula immediately before the application of Riboflavin. Subsequently, the CXL throughout iontophoresis was performed as an epi-on technique as explained before, and a contact lens was placed onto the ocular surface and removed after one week [17].

Postoperatively, an ophthalmic gel of 0.15% sodium hyaluronate, 1% xanthan gum, and 0.3% netilmicin (Xanternet Gel, SIFI, Catania, Italy) was prescribed 6 times a day until complete epithelial regrowth (epithelial integrity was assessed with fluorescein staining). In patients who underwent epi-on iontophoresis CXL, in whom a contact lens was not used, dexamethasone 21-phosphate 0.15% drops were prescribed 4 times a day for 10 days and 0.15% sodium hyaluronate, Riboflavin, L-Leucin, L-Prolin, L-Glycin, and L-ysin drops (Ribolisin free, Sooft) 6 times daily for 45 days. In patients who underwent epi-off iontophoresis CXL, after contact lens removal, dexamethasone 21-phosphate 0.15% drops were prescribed 4 times a day for 10 days and 0.15% sodium hyaluronate, Riboflavin, L-Leucin, L-Prolin, L-Glycin, and L-Lysin drops (Ribolisin free, Sooft) 6 times daily for 45 days. In addition, all patients received oral amino acid supplements (Aminoftal; Sooft) for the first 7 postoperative days in effort to promote epithelial healing.

### 2.2. Ophthalmic Examination

Each patient underwent clinical preoperative examination, including manifest refraction, Slit Lamp Examination, and corneal topography (Pentacam, Oculus, Wetzlar, Germany), recorded at 1 month, 3 months, 6 months, 12 months, and 24 months after surgery. Several topographic parameters were analyzed including Kmax; Mean K; Steep K; Flat K; True Net Power; Mean Corneal Thickness; and Thinnest corneal point. Corneal Tomography was also focused on the presence/absence of inflammatory cells and the activation of corneal keratocytes which is related to the development of fibrosis [18,19,20]. The considered Pentacam parameters provide information about corneal refractive power, calculated thanks to the Sheimpflug principles. The Scheimpflug system consists of a rotational slit camera producing a three-dimensional model of the anterior segment of the eye from 138,000 elevation points. Studying this model, the operator is able to measure the dioptric corneal power in each point and axis of the anterior segment (Kmax, Mean K, Steep K, Flat K) and measure corneal power in total (true Net Power) by computing the power of the anterior and posterior corneal surface without taking in consideration corneal thickness. The study of Corneal Thickness profile enables one to collocate the reduction in corneal depth in the corneal profile, allowing one to correlate them with the highest and steepest points of the cornea.

Symptoms evaluation was performed before and one month after surgery using the Ocular Surface Index (OSDI) questionnaire.

### 2.3. Statistical Analysis

The statistical analysis was carried out using IBM SPSS 25 for Mac (SPSS Inc., Chicago, IL, USA). The averages were compared by an ANOVA test unchanged according to Bonferroni. In all the statistical tests, 5% was used as a significant value.

## 3. Results

A total of 64 eyes of 49 patients underwent CXL, 30 using the epi-off technique, and 34 eyes the epi-on (Table 1). The mean age of patients was 26 ± 4.2. In the epi-off group, CDVA converted in logMAR were at baseline 0.3 ± 0.1 and improved to 0.22 ± 0.14 at 2 years follow-up clinical examination. In the epi-on group, the visual acuity values converted in logMAR improved from 0.22 ± 0.1 at baseline to 0.09 ± 0 at the last follow-up. We found no statistically significant differences between the two groups (*p* = 0.9).

The statistical analysis performed (Table 2) showed no statistically significant difference between Kmax, Mean K, Flat K, Steep K values before and 24 months after surgery in both groups.

The mean K at baseline was 47.20 ± 2.90 D and 48.00 ± 2.50 D in the epi-off and epi-on groups, respectively. After two years of follow-up, these two values remained unchanged, 47.16 ± 3.15 D in the epi-off group and 48.82 ± 4.06 D in the epi-on group, with no statistically significant differences between the groups (*p* = 0.57).

Steep K and flat K at baseline were 47.75 ± 3.20 D and 44.62 ± 2.63 D, respectively, in the epi-off group. After two years of follow-up, these values remained unchanged at 47.55 ± 3.12 D and 45.02 ± 2.03 D with no statistically significant differences between the two groups (*p* = 0.69). In the epi-on group, Steep K and flat K at baseline were 46.75 ± 3.87 D and 45.62 ± 2.09 D, respectively. After two years of follow-up, these values remained unchanged at 47.05 ± 2.82 D and 45.42 ± 1.83 D with no statistically significative differences between the two groups (*p* = 0.44).

On the contrary, the Kmax mean values were 53.6 ± 1.4 D for the epi-on technique and 58.1 ± 9.2 D in the epi-off group at baseline and of 55.9 ± 0.7 D and 56.2 ± 1.8 D, respectively, with a statistically significant difference (*p* < 0.05) between the epi-on and epi-off groups’ pachymetry before and after 24 months follow up.

After 2 years, the mean corneal thickness was not significantly changed in both groups, from 491.5 ± 1.3 μm to 467.41 ± 19 μm for the epi-on group (*p* = 0.7) and from 475.41 ± 21.9 μm to 497 ± 27 μm for the epi-off group (*p* = 0.7).

**Figure 1:**
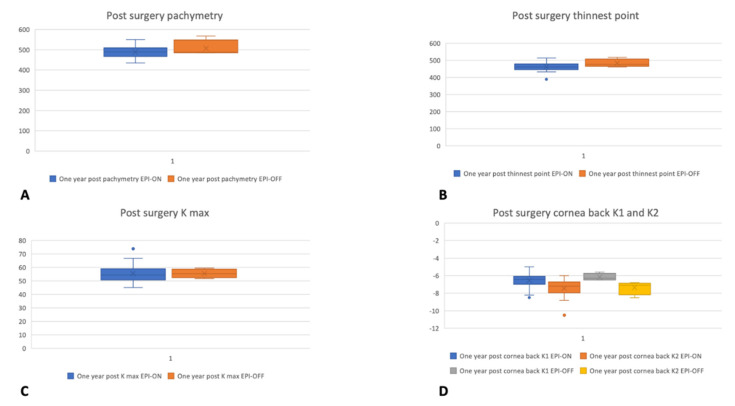
Kmax (**A**), Pachymetry (**B**), thinnest point (**C**), and cornea back (**D**) topographic outcomes before surgery and after one year of follow-up in the epi-on and epi-off groups.

Contrarily, a statistically significant difference (*p* < 0.05) of the topographically thinnest point immediately post-surgery and 24 months after surgery emerged. Thickness average values were higher in patients who underwent epi-off surgery; indeed, the mean pre-surgery thinnest point was 478 μm, and after 24 months of follow-up, it was 518 μm (Figure 1). Particularly, no significant changes in both groups, from 466.2 ± 15.5 μm to 467.41 ± 19 μm for the epi-on group (*p* = 0.7) and from 475.41 ± 21.9 μm to 497 ± 27 μm (*p* = 0.7) in the epi-off group, occurred after 2 years of follow-up.

The ANOVA Univariate analysis highlighted no statistically significant differences in the Average Pachymetry and the Topographically Thinnest Point between groups during the follow-up period; this is best displayed in Figure 1. Moreover, statistically significant differences between Kmax values before surgery and after 1 month (*p* < 0.008), 3 months (*p* > 0.001), and 6 months of the follow-up period (*p* > 0.002) were identified. On the one hand, during the follow-up period, Kmax values were on average higher than time zero. On the opposite side, the Kmax values came back to the before surgery values at the last follow-up after 24 months.

Lastly, the data collected from the OSDI© questionnaire highlighted at the baseline a score of 4.58 ± 1.18 and 4.89 ± 1.32, respectively, in the epi-off and epi-on groups. After 2 years of follow up, the score increased to 13.65 ± 2.15 in epi-off group (*p* < 0.01) and 11.62 ± 2.12 in epi-on (*p* < 0.04). We also found a statistically significant difference between the two groups (*p* < 0.02).

## 4. Discussion

Corneal collagen cross-linking stops the progression of keratoconus through new covalent bonds between collagen fibers, which increase the strengthens and the rigidity of the cornea by more than 300% [21]⁠, with a parallel improvement in both the refractive and topographic features of treated corneas [22,23]. Wollensak et al. [15], in a non-randomized pilot study conducted on 22 patients, described a clinical technique to halt the KC progression in all treated eyes. The study highlighted a halt in the progression of all treated eyes. Thereafter, several studies confirmed the Wollensak results, demonstrating the efficacy of corneal cross-linking in halting the progression of keratoconus. One of the largest trials including 241 eyes was conducted by Raiskup-Wolf et al. [10], who emphasized that the treatment was able to decrease the steepness of the cone, improving subjective visual symptoms, and, consequently, uncorrected (UDVA) and corrected (CDVA) distance visual acuities [16].

Most of the complications related to standard CXL are associated with epithelial removal, i.e., postoperative pain, stromal haze, infection, and corneal melting [24,25]⁠. Other alternatives, avoiding total removal of the epithelium, have been suggested in order to improve safety, including partial de-epithelialization, epithelial disruption, the creation of an epithelial flap (Epi-Flap CXL), and several trans-epithelial CXL techniques. The potential advantages of trans-epithelial techniques are reduced treatment time, prevention of slow visual recovery, and less risk of serious side effects and complications (postoperative pain, infection) while maintaining the same efficacy of the standard epi-off procedure [26,27,28,29,30,31]. Shalchi et al. [32]⁠ compared the results of standard epi-off CXL with a metanalysis of 45 papers referred on transepithelial CXL with a total of 5 papers in the management of progressive keratoconus. The majority of studies on standard CXL have shown a reduction in the maximum simulated keratometry, whereas transepithelial corneal CXL did not halt keratoconus progression in about 75% of cases within 1 year [33]⁠. Moreover, the shortening riboflavin delivery time from 30 to 5 min is not negligible. Our study supports the concept that CXL with Iontophoresis is effective in halting the progression of keratoconus without the side effects of epithelial removal, encountered the performing standard epi-off CXL procedure. In addition, the comparison between epi-on and epi-off techniques, both with the iontophoretic corneal soak in our series, confirmed their efficacy in halting keratoconus progression. With the epi-off iontophoretic approach, we guaranteed a deep penetration of riboflavin with a shorter time of delivery. Moreover, epithelial debridement and repair can determine a better quality of vision with a reduction in higher-order aberration thanks to a better distribution of the epithelium from the base to the apex of the cone [34] Furthermore, unlike standard treatment, we found a great recovery of the corneal shape and thickness in patients who underwent epithelium removal before iontophoresis CXL. These outcomes could be related to the epithelium reparative process that regularizes the corneal surface [35].

In addition, keratocyte repopulation is related to stromal growing factors influencing the correct orientation of stromal lamellae and nerve regeneration. In fact, survived keratocytes were stimulated by stromal fibroblast through neurotrophic and inflammatory factors [18,19,20,36]. This regenerative process requires new structural and enzymatic protein to be available. The obtainability of amino acids becomes essential for the correct stromal and nerve regeneration. As a result, the topical and oral supplementation of essential amino acids may contribute to a better and faster corneal restoration [37].

Standard Dresden protocol CXL (S-CXL) is used as a treatment of progressive keratoconus in pediatric and adult patients, and its efficacy is widely reported in numerous studies [38,39,40,41]. Nevertheless, this technique presents two disadvantages: It is time-consuming (about 60 min for each surgery) and requires epithelial debridement. With the introduction of transepithelial CXL and subsequently of Iontophoretic cross-linking (I-CXL), the same outcomes were achieved with shorter surgery time and no corneal manipulation. Cantemir et al. [42], with a 3-year follow-up study, showed that I-CXL is not inferior to S-CXL for stopping the progression of keratoconus with faster recovery of visual acuity. Jouve et al. [43] reported that I-CXL halted the progression of keratoconus more efficiently compared to S-CXL with regard to the flattening effect. The introduction of I-CXL with epithelial debridement (SI-CXL) was designed as an improvement in S-CXL. The main outcome of the study underscored the non-statistically significant difference between the protocols about visual acuity, topographic indices, keratometry, and OSDI after 2 years of follow-up. SI-CXL induced less corneal thinning and a significantly higher reduction in higher-order aberrations and coma with a better visual outcome. In 2019, Vinciguerra et al. [35] compared S-CXL, I-CXL, and SI-CXL focusing on refractive, topographic, tomographic, and aberrometric outcomes demonstrating that SI-CXL was not inferior compared to the other techniques. Cifariello et al. [44] were the first to analyze OSDI© (Ocular Surface Disease Index) differences in patients who underwent CXL. According to their previous study, we evaluated the degree of ocular discomfort in patients treated with I-CXL and SI-CXL. The results showed an increase in OSDI score to 13.65 ± 2.15 in the epi-off group (*p* < 0.01) and 11.62 ± 2.12 in the epi-on group (*p* < 0.04) at 2 years of follow-up. A statistically significant difference between the two groups (*p* < 0.02) was found.

## 5. Conclusions

In conclusion, we brought to light that both techniques are valid in long-term corneal stabilization. Moreover, the mechanical removal of the corneal epithelium would still seem the most valid method to obtain a better saturation of corneal stroma with the photosensitizer and better postoperative results. The advantage of the new iontophoresis epi-off cross-linking technique could be the faster corneal imbibing related to the iontophoretic soak and the better visual acuity with a reduction in higher-order aberration linked to epithelium debridement.

## Figures and Tables

**Table 1:** Patients’ Demographics.

	Frequency	Percentage
Gender		
Male	32	65.30%
Female	17	34.69%
Age		
Mean age of all patients	26 ± 4.2	
Mean age of Epi-on group	25 ± 5.6	
Mean age of Epi-off group	27 ± 6.3	
Eyes		
Treated	64	65%
Untreated	34	34.70%
Technique		
Epi-on	34	53%
Epi-off	30	47%

**Table 2:** Postoperative Outcomes.

	T0	T3	T6	T12	T24	ANOVA
Differences with Respect to Preoperative CDVA (Logmar)						
Epi-on		0.22 ± 0.1	0.15 ± 0.2	0.09 ± 0	0.09 ± 0	0.9
Epi-off		0.3 ± 0.1	0.3 ± 0.1	0.22 ± 0.14	0.22 ± 0.14	
Kmax (D)						
Epi-on	53.6 ± 1.4	54.2 ± 0.8	53.2 ± 3.5	55.6 ± 0.7	55.9 ± 0.7	0.96
Epi-off	58.1 ± 9.2	58.8 ± 1.76	58.8 ± 7	55.9 ± 1.8	56.2 ± 1.8	
Mean K (D)						
Epi-on	48.00 ± 2.50	47.96 ± 3.25	48.2 ± 1.75	48.96 ± 2.80	48.82 ± 4.06	0.57
Epi-off	47.20 ± 2.90	46.89 ± 4.15	47.1 ± 1.75	47.00 ± 2.40	47.16 ± 3.15	
Steep K (D)						
Epi-on	46.75 ± 3.87	46.55 ± 2.97	46.15 ± 4.07	45.75 ± 4.17	47.05 ± 2.82	0.69
Epi-off	47.75 ± 3.20	47.45 ± 2.79	47.15 ± 3.00	47.62 ± 4.20	47.55 ± 3.12	
Flat K (D)						
Epi-on	45.62 ± 2.09	44.92 ± 2.09	45.23 ± 3.12	45.11 ± 2.65	45.42 ± 1.83	0.44
Epi-off	44.62 ± 2.63	44.99 ± 2.74	45.10 ± 3.00	44.92 ± 1.68	45.02 ± 2.03	
True net power (d)						
Epi-on	2.8 ± 1.7	3.2 ± 1.2	2.5 ± 0.1	3.2 ± 0.1	3.4 ± 0.4	0.67
Epi-off	4.5 ± 2.8	5 ± 0.6	3.7 ± 0.07	5.6 ± 0.7	5.7 ± 1.2	
Mean corneal thickness (µm)						
Epi-on	491.5 ± 11.3	484.8 ± 24	495.9 ± 19	489.5 ± 13.4	490.5 ± 13.4	0.7
Epi-off	500.5 ± 22.6	481 ± 24.5	499.7 ± 63.6	513 ± 3.1	515 ± 4	
Thinnest point (µm)						
Epi-on	466.2 ± 15.5	463 ± 26.1	473.2 ± 4.2	463 ± 22.6	467 ± 19	0.72
Epi-off	475.8 ± 21.9	452.1 ± 19	463.2 ± 31.8	491 ± 12	497 ± 27	
OSDI score						
Epi-on	4.89 ± 1.32	5.78 ± 1.2	6.89 ± 0.1	9.87 ± 2.5	11.62 ± 2.12	0.98
Epi-off	4.58 ± 1.18	8.01 ± 0.6	9.12 ± 0.23	12.45 ± 1.32	13.65 ± 2.15	

T0 = baseline; T3 = 3 months postoperatively; T6 = 6 months postoperatively; T12 = 12 months postoperatively; T24 = 24 months postoperatively; ANOVA= *p* value of the difference between groups; CDVA = corrected distance visual acuity; CXL = corneal cross-linking; I-CXL =transepithelial iontophoresis; I-SCXL = iontophoresis with epithelial debridement.

## Data Availability

The data presented in this study are available on request from the corresponding author.
